# Effectiveness of Social Virtual Reality Training in Enhancing Social Interaction Skills in Children With Attention-Deficit/Hyperactivity Disorder: Protocol for a Three-Arm Pilot Randomized Controlled Trial

**DOI:** 10.2196/48208

**Published:** 2023-09-18

**Authors:** Ka Po Wong, Jing Qin

**Affiliations:** 1 Department of Applied Social Sciences The Hong Kong Polytechnic University Hong Kong China (Hong Kong); 2 Centre for Smart Health School of Nursing The Hong Kong Polytechnic University Hong Kong China (Hong Kong)

**Keywords:** attention-deficit/hyperactivity disorder, social interaction skills, executive functioning, emotional control, 3-arm randomized controlled trial, ADHD, attention deficit, hyperactive, hyperactivity, randomized, RCT, social interaction, social interactions, social skills, child, children, youth, pediatric, pediatrics, VR, virtual reality, childhood, neurodevelopmental

## Abstract

**Background:**

Attention-deficit/hyperactivity disorder (ADHD) is one of the most common neurodevelopmental disorders among children. Children with ADHD have challenges in understanding social cues and behavioral problems when entering a social setting. Virtual reality (VR) has been applied to improve cognitive behaviors in children with ADHD. Previous studies have not adopted VR to improve social interaction competence and appropriateness in children with ADHD. VR offers a more effective alternative to therapeutic strategies for children with ADHD.

**Objective:**

This study aims to examine the feasibility and effectiveness of social VR training in enhancing social interaction skills compared to traditional social skills training in children with ADHD. We hypothesize that participants in the social VR training group are likely to perform better on social interaction skills than those in the traditional social skills training group.

**Methods:**

In this nonblinded, 3-arm randomized controlled trial (RCT), 90 participants with ADHD recruited from the community will be randomized 1:1:1 to the social VR intervention group, traditional social skills training group, or waitlist control group. The child psychiatrist will conduct assessments for each participant at baseline and after the intervention. The Social Skills Rating Scale–Parent will be used to assess the social interaction skills of the participants before and after the intervention. Participants in the social VR intervention group and traditional social skills training group will receive twelve 20-minute training sessions for 3 weeks. The participants in the waitlist control group will receive no training. The primary outcome measure is training acceptability and compliance. The secondary outcome measures are the child psychiatrist's assessment and the Social Skills Rating Scale–Parent before and after the intervention. Another outcome measure is the Behavior Rating Inventory of Executive Function and Attention. Differences in the scale scores will be examined using a *t* test and an *F* test.

**Results:**

This study is set to commence in the fourth quarter of 2023. It is anticipated that participants in the social VR intervention group will exhibit superior social interaction skills than those in the traditional social skills training group.

**Conclusions:**

To our knowledge, this RCT is the first study examining the feasibility and effectiveness of a social VR-based intervention for enhancing the social interaction skills of children with ADHD in Hong Kong. The VR-based social skills training is expected to provide a safer and more effective environment for children with ADHD to learn than the traditional approach. This study can lead to a full-scale RCT.

**Trial Registration:**

ClinicalTrials.gov NCT05778526; https://clinicaltrials.gov/study/NCT05778526

**International Registered Report Identifier (IRRID):**

PRR1-10.2196/48208

## Introduction

### Background

Attention-deficit/hyperactivity disorder (ADHD) is one of the most common neurodevelopmental disorders frequently diagnosed in school-aged children. It is characterized by pervasive symptoms of inattention, hyperactivity, and impulsivity. There are 3 main types of ADHD: inattentive type, hyperactive-impulsive type, and combination type. ADHD impacts the children’s emotions, behaviors, and ability to learn [1]. The hyperactivity and inattention levels of children with ADHD are noticeably higher than expected, causing distress or problems at home, at school, and with peers. The global prevalence rate of ADHD is about 5% to 7% [2]. According to the data from the Family Council in Hong Kong [3], there are 14,580 students with ADHD in Hong Kong, which is the second-highest number among the 9 types of special education needs.

Executive functions refer to a set of cognitive processes that enable an individual to plan, organize, manage, and execute a task, which includes working memory, cognitive flexibility, and inhibitory control [4]. Children with ADHD’s executive functions can be delayed by up to 32% compared with typically developing peers [5]. Such delay can have tremendous impacts on the daily lives of individuals with ADHD [6]. For instance, inattention symptoms cause these children to have difficulty concentrating and listening to others, become distracted by the environment, and become overwhelmed [6]; hyperactivity symptoms cause high levels of distraction, sharing scattered thoughts, and excessive talking; impulsivity symptoms cause inappropriate initiation of conversations and becoming aggressive [4]. These children have relatively poor self-control, inhibitory control, emotion regulation, attention, and working memory [5]. Hence, children with ADHD have challenges in understanding social cues and behavioral problems when entering a social setting. They have difficulty comprehending some nonverbal expressions (body language, eye contact, and facial expressions) and spoken communication (pitch, speed, tone, and volume), making it difficult to listen to others and share their thoughts appropriately when interacting with them [7]. The social skill deficit causes it to become difficult to build friendships or even peer rejection [7]. To further avoid peer rejection, some of these children may avoid social interaction. Thus, it is critical to assist these children in developing appropriate social interaction skills.

ADHD has been associated with a wide range of detrimental impacts on those affected and imposes a severe financial burden on families and society [8,9]. The social problems faced by children with ADHD are associated with strained family relationships and social isolation from peers, which, in turn, can adversely affect emotional well-being, as some of them may experience both anxiety and depression [10]. Several studies reported that the economic burden of raising a child with ADHD was 5 to 6 times that on their counterparts, and the parents of children with ADHD tended to be fired and have low work efficiency [8,9,11]. Therefore, adequate and appropriate treatments should be provided to alleviate their behavioral issues and high health care costs. The commonly used social skills training tools among children with ADHD are traditional instructions, modeling, role-play activities, and behavior rehearsal [12]; however, these methods are limited to time and space and are short of real-life scenarios [13].

Virtual reality (VR) has emerged in plenty of domains as a promising tool for therapy and rehabilitation. Numerous studies adopted VR in improving cognitive behaviors in children with ADHD; however, the focuses of these studies were cognitive functioning in the context of daily living, attentional performance, and impulsivity [14-16]. None of these studies used VR to improve social interaction competence and appropriateness in children with ADHD. Furthermore, VR provides controllable immersive environments to engage children, sustain their attention, and allow them to effectively gain skills in a safer environment [17]. VR systems generally consist of a head-mounted display and screen so that the therapy can be conducted in a relatively low spatial limitation. Although no study has used VR to investigate social interaction skills in children with ADHD, previous studies have advocated the use of VR-based social skills training for children with autism spectrum disorder (ASD) [18-20]. Most of the studies about children with ASD also included some real-life scenarios—for example, classrooms, transportation, and supermarkets—in their social skills training program. This implies that children can develop social skills in a safe and controlled setting that can be designed in accordance with their needs [21]. VR can create different real environments for children to practice in repeatedly [21]. VR can provide instant feedback, which can help children recognize the appropriateness of their social interaction in practice [22]. The differences in social skills between children with ADHD and those with ASD are that the former are easily distracted, avoid or dislike concentrating on one thing, and are more likely to interrupt others when they are speaking, while the latter are less aware of others around them, overly attached to one object that they like, and find it difficult to give meaning to their speech [23]. Due to these discrepancies, the design of a social VR intervention in this study will be integrated with more instantaneous events, objects, and avatars in each scenario to improve the problem of distraction among children with ADHD. In addition, social VR, which allows more than 1 user to appear as avatars in a lifelike environment and connect with other users in real time, will be adopted in this study instead of VR to enhance engagement and interaction among children with ADHD.

Considering the prevalence and advantages of social VR, we plan to develop a social VR training program that contains 3 real-life scenarios and compare the effects of social VR training and traditional social skills training on social interaction skills, inhibitions, emotional control, behavioral and emotional difficulties, and conduct disorders for children with ADHD in Hong Kong receiving no training as a control.

Cultivating sufficient and appropriate social interaction skills for children with ADHD is a vital step to assist them in integrating into the community. VR technologies offer a more effective alternative to therapeutic strategies for children with ADHD. However, no previous study has adopted social VR to assess the competence and appropriateness of social interaction in children with ADHD, and no randomized controlled trial (RCT) has compared the effectiveness of social VR training and traditional social skills training on enhancing social interaction skills in children with ADHD. Traditional social skills training for children with ADHD lacks the provision of real-life scenarios and is limited by time and space. Social VR allowing real-time connection and interaction between patients and the virtual instructor has been absent in previous studies.

To fill these research gaps, we proposed conducting a 3-arm RCT to answer the research question of whether social VR training has higher effectiveness than traditional social skills training in enhancing the social interaction skills of children with ADHD in Hong Kong. The findings of this study are expected to unveil the clinical impacts and compliance and acceptability of social VR training for enhancing social interaction skills among children with ADHD.

### Aims and Hypotheses

The study targets children diagnosed with ADHD and aims to (1) develop a social VR intervention, (2) investigate its effectiveness in improving the social skills of the children compared to traditional social skills training, and (3) evaluate the acceptability of and compliance with social VR training for enhancing social interaction skills. We hypothesize that participants in the social VR training group may perform better on social interaction skills than those in the traditional social skills training group. Participants in the waitlist control group are expected to show no change in social interaction skills compared with the 2 intervention groups.

## Methods

### Overview

**Figure 1:**
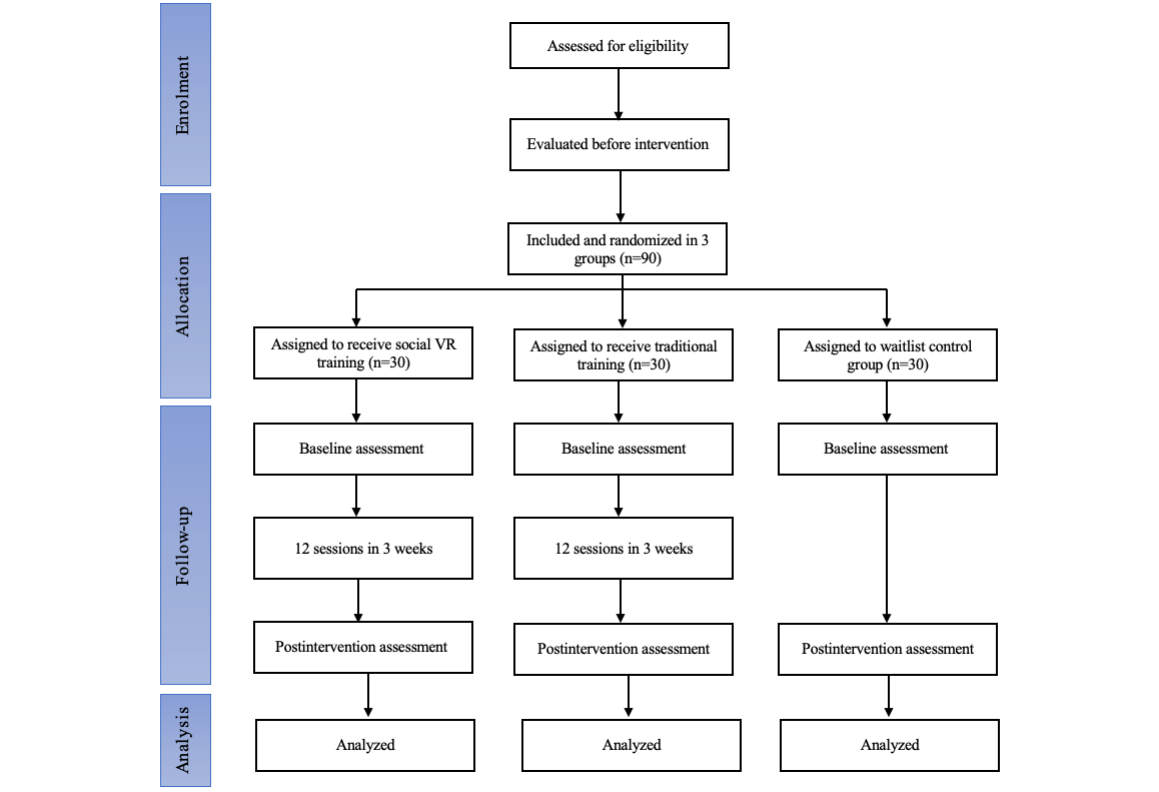
Flowchart of the study.

This study will be a 3-arm RCT comparing the effects of a social VR intervention with those of traditional social skills training on social skills and executive functioning of children with ADHD. Participants in the social VR intervention group and those in the traditional social skills training group will receive 12 training sessions for 3 weeks (4 sessions per week), and those in the waitlist control group will be asked to retain their usual lifestyles for 3 weeks. The overall design of the study is illustrated in Figure 1.

### Participant Selection Criteria

Individuals who meet the inclusion criteria will be recruited. The criteria are as follows: (1) age between 6 and 12 years; (2) being ethnic Chinese; (3) residing in Hong Kong; (4) having received a diagnosis of ADHD by a Child Assessment Service in Hong Kong or via private practice; (5) being stable on pharmacological or psychological treatment (or both) for ADHD 8 weeks before baseline; (6) no initiation or change in pharmacological treatment for ADHD during the intervention period; (7) ability of the child and at least one of the child’s parents or legal guardian to read Chinese and speak and listen to Cantonese; and (8) willingness to provide informed consent by both participants and one of their parents or legal guardian.

Exclusion criteria are as follows: (1) comorbid autism; (2) an estimated IQ lower than 85 (determined using the Wechsler Intelligence Scale for Children–Fourth Edition; Hong Kong); (3) ASD (previously diagnosed by health care professionals); (4) comorbid acute psychiatric disorder (previously diagnosed by health care professionals); (5) a comorbid severe physical disability (eg, blindness or deafness); and (6) having a learning disability in understanding and using spoken Chinese, calculating, and motor coordination.

### Sample Size

Teare et al [24] and Whitehead et al [25] indicated that studies with a sample size of 20 participants per arm have at least 80% power and 90% confidence. A pilot study by van der Oord et al [26] involved 40 participants (20 in the control group and 20 in the intervention group) to examine the efficacy of computerized executive functioning remediation training with game elements in children with ADHD. The sample size of this pilot RCT is more than the recommended sample sizes and that of the published pilot studies; hence, a proposed sample size of 90 (30 per arm) could provide more reliable results. To obtain a statistical power of 90% at a significance level of .05 and assume that the maximum dropout rate is 50%, we aim to recruit 30 participants in the social VR group, 30 in the traditional social skills training group, and 30 in the waitlist control group (ie, a total of 90 participants).

### Ethical Considerations

Ethical approval for the study has been obtained by the Human Subjects Ethics Application Review Committee of The Hong Kong Polytechnic University (HSEARS20221221003). Informed consent will be sought from the participants and their parents or legal guardians.

### Procedure

#### Recruitment

Participants will be recruited from nonprofit organizations and community centers. The inclusion and exclusion criteria will be listed on the recruitment poster. Interested participants or parents who provide written informed consent will be further evaluated for inclusion and exclusion criteria by a clinical psychologist or a student with a master's degree in clinical psychology. Participants who meet the inclusion criteria will be invited to perform an IQ test at the university at baseline.

#### Randomization

Eligible participants will be randomized to the social VR group, traditional social skills training group, or waitlist control group. A computer-generated randomizer will be used to generate the random allocation list. Randomization will be undertaken by another research assistant not directly involved in the study. A number generated by the computer will be assigned to each eligible individual who will be randomly allocated to the 3 different groups based on the number. Parents or legal guardians will receive an email and a telephone call with a notification regarding allocation to groups 1-3.

### Social VR Intervention

This social VR intervention is being developed to enhance the social interaction skills of children. The participants will wear a head-mounted display for the social VR intervention. Each session of the social VR intervention lasts for a maximum of 20 minutes to ensure that the participants focus on the intervention and to prevent causing any physical effect [27]. The duration will be adjusted depending on the emotions of the participants during the intervention. The social VR intervention will mainly help the participants enhance their social interaction skills and executive function. The intervention contains three real-life virtual scenarios, including (1) a classroom and playground, (2) mass transit railway, and (3) a supermarket and restaurant. One scenario will be adopted in each session. The sequences of the scenarios used in each session will be the same for all participants. During the social VR intervention, an research assistant will also appear as one avatar in the scenario to guide the participants to complete a series of tasks. Each intervention session will be conducted in a classroom independently for each participant.

### Traditional Social Skills Training

An instructor with a background in education, psychology, or social work will teach the participants social interaction skills through traditional instructions and role-play activities. Four modules will be covered in the 3-week training: (1) how to introduce yourself and basic social skills, (2) how to listen to others, (3) how to share with others, and (4) learn to understand how people feel and how to empathize. These modules have been applied in many studies [12,28]. The content of this training will be as similar as possible to the social VR training. The training lasts 20 minutes, which depends on the participants’ emotions. Each training session will be conducted in a classroom independently for each participant.

### Waitlist Control Group

As in Beck et al’s [29] study, the participants in this group will receive no training and they can participate in the social VR training after the intervention period. To ensure consistency in the experiment, the participants are not allowed to initiate or change their pharmacological treatment during the 3-week intervention period.

### Outcomes Measures

#### Questionnaires

The parents or legal guardians of the participants will complete a structured questionnaire at baseline and immediately after the last session. Demographic information, including age, gender, medication, ADHD symptoms, and game experience will be recorded at baseline. The questionnaires include questions about social skills and executive functioning. The satisfaction of the participants and their parents or legal guardians with the training program will be assessed at week 3. VR sickness of the participants in the social VR group will be evaluated after each session. The clinical psychologist who evaluated the performance of the participants will be blinded to the treatment condition. The questionnaires will include the scales described in the following paragraphs.

#### Primary Outcome Measure: Assessment of Training Acceptability and Compliance

The attendance of the participants during the training will be recorded. To verify the validity of the findings, training-nonadherent participants will be excluded, which will be stated in the consent form. Absence in 3 or more training sessions will be considered nonadherence. The participants will be asked about their willingness to partake in this kind of training with a 7-point Likert scale question, which is used to reflect the acceptability of the intervention. The retention rate should be at least 85% according to a prior feasibility RCT [30].

Compliance with the training program will be evaluated by the communication log, in which the parents record their children’s daily activity and emotions in the 3-week intervention period. The participants’ parents will be reminded to attend the training by telephone in the first 4 training sessions and by WhatsApp, Signal, or WeChat in the fifth and eighth training sessions. No follow-up reminder will be provided in the remaining 4 sessions. These data will be used to evaluate their compliance.

#### Secondary Outcome Measures

##### Social Skills Rating Scale–Parent

The social skills of the children will be assessed using the Chinese-translated Social Skills Rating Scale–Parent (SSRS-P), which will be scored by the participants’ parents before the first session and after the last session. This scale consists of 3 subscales, including self-control, assertiveness, and initiative and cooperation, with a total of 31 items. A 3-point Likert scale (never, sometimes, or often) is used to score. The SSRS-P is a validated instrument that has been commonly adopted in clinical trials of psychiatric and neurological disorders.

##### Child Psychiatrist’s Assessment

One child psychiatrist will score the basic social skills of the participants in 15-minute tasks before the first session and after the last session to obtain an objective evaluation. The basic social skills assessment is adapted from the scale of Riggio [31]. Broken blinding is unavoidable in the child psychiatrist’s assessment. To minimize unblinding bias during the assessment, each participant will be identified by a case number.

##### Behavior Rating Inventory of Executive Function

The Chinese-translated Behavior Rating Inventory of Executive Function (BRIEF), used to assess executive functioning in children, will be scored by the participants’ parents before the first session and after the last session. The subscales of inhibitions (16 items) and emotional control (10 items) will be adopted in this study. A 3-point Likert scale (never, sometimes, or often) is used to score.

##### Satisfaction

Satisfaction is measured by a question rated with a 7-point Likert scale: “What grade would you give to this training program?” Both participants and parents or guardians have to answer the question.

##### Process Evaluation

Process evaluation of the training will be assessed by investigating (1) the recruitment rate, (2) participant completion, (3) side effects of training measured by participants’ sickness or physical discomfort in the VR environment, and (4) safety assessment including adverse events and reasons for withdrawal.

- Recruitment rate: the recruitment rate is at least 48.5% of all referrals according to a prior feasibility study [32].
- Participant completion: participants attending at least 9 of the 12 (75%) sessions are considered to have completed the program [33].
- Simulator Sickness Questionnaire: the Simulator Sickness Questionnaire measures the motion sickness or physical discomfort of participants in a VR environment. Nine items will be measured, including discomfort, fatigue, headache, eyestrain, sweating, nausea, difficulty concentrating, blurred vision, and dizziness, with yes-or-no questions for each item.
- Assessment of safety: (1) adverse events: participants’ parents or guardians will be asked, using open-ended questions, whether the participants experienced any adverse events during training. The severity and relationship of the adverse event to the intervention will be documented and investigated. (2) Reasons for withdrawal: when a participant withdraws before completing the study, the reasons for withdrawal will be recorded.

### Statistical Analysis

All statistical analyses will be performed using SPSS software (version 26.0; IBM Corp) and are 2-sided with a level of significance of <.05. Demographic information of the participants will be summarized with frequency and percentage values for categorical variables. The continuous variables will be summarized with mean and SD values. The change from baseline to week 3 within each arm will be tested using *t* tests. To examine the between-group difference in measure outcomes from baseline to week 3, repeated analyses of covariance with adjustment of baseline characteristics will be conducted. To evaluate improvements during the intervention period among the 3 groups, *F* tests will be performed with primary and secondary outcome measures at the beginning of the first session and at the end of the last session. An intention-to-treat analysis will be performed. This analysis is considered the most appropriate approach to analyze RCT data by the highest-quality studies that have used the CONSORT (Consolidated Standards of Reporting Trials) standard [34].

## Results

Significant changes are expected to be noted in the mean values of the secondary outcomes, namely basic social skills as assessed by child psychologists, and social skills and executive functioning as assessed by parents, using the intention-to-treat principle. Conducting this pilot RCT will determine the feasibility and potential of social VR interventions in improving social interaction skills in children with ADHD.

## Discussion

### Anticipated Findings

To the best of our knowledge, this RCT is the first study examining the effectiveness of a social VR intervention for enhancing the social interaction skills of children with ADHD in Hong Kong. The study will contribute to evidence-based practice by testing the effectiveness of social VR training versus traditional social interaction skills training for children with ADHD. It may determine a causal relationship between social VR training and traditional social skills training on social interaction skills and executive functioning. The results of this study will provide clinical psychologists with an effective means to improve the social interaction skills of children with ADHD in Hong Kong. Social VR used in this study strengthens the attractiveness of the training and the interaction and communication between patients and guiders and the real-life situations. Considering their high availability and accessibility, the cost and time of developing the social VR programs would be worthwhile if they could be promoted among more children with ADHD to enhance their social interaction skills with limited human resources. In addition, these findings will facilitate the development of a social VR training program that is customized for children with ADHD in Hong Kong, resulting in the improvement of these children’s social skills and psychological well-being; this will be beneficial in integrating them into society. There is potential for the government to set up an accreditation scheme to enhance the social VR training to cater to the children with special education needs in various settings, including special schools, integrated schools, community centers, and social support groups.

### Conclusions

This pilot RCT has the potential to enhance the understanding of the feasibility and effectiveness of using social VR to improve social skills in children with ADHD. This will help determine whether the use of social VR is acceptable for children with ADHD and understand which approach (ie, social VR or traditional social skills training) is most likely to achieve better outcomes. It will also determine the feasibility of a larger trial.

## Appendix

Multimedia Appendix 1 External peer review report.

## Abbreviations

ADHD: attention-deficit/hyperactivity disorder
ASD: autism spectrum disorder
BRIEF: Behavior Rating Inventory of Executive Function
CONSORT: Consolidated Standards of Reporting Trials
RCT: randomized controlled trial
SSRS-P: Social Skills Rating Scale–Parent
VR: virtual reality
