# Supplementary material for: Effectiveness of Social Virtual Reality Training in Enhancing Social Interaction Skills in Children With Attention-Deficit/Hyperactivity Disorder: Protocol for a Three-Arm Pilot Randomized Controlled Trial
Source: JMIR Res Protoc. 2023 Sep 18;12:e48208. doi: 10.2196/48208 (PMC10546265; doi:10.2196/48208)
Supplement: Multimedia Appendix 1 [file resprot_v12i1e48208_app1.pdf]

The Hong Kong Polytechnic University

School of Nursing

School Research Committee

Assessment Form

Project title: Effectiveness of social virtual-reality on enhancing social interaction skills in children with attention-deficit/hyperactivity disorder: a three-arm pilot randomised controlled trial

Section A: Written comments

Please comment on the strength and /or weaknesses of the proposal, considering:

a) Originality and impact
b) Methodology proposed
c) Outcomes and data analysis
d) Ethical considerations
e) Research capability

This information will be released to the applicant(s) to help them improve the proposal. Please use additional sheet if space is insufficient)

This is a promising proposal in addressing the problems of social interaction competence and appropriateness in children with ADHD with the use of virtual reality technology. Compared with the commonly used social skill training such as tradidactic instructions, modelling, role-play activities and behaviour rehearsal, the VR settings provide controllable immersive environments to engage children, sustain their attention and allow them to effectively exercise their skills in a safer environment, and as well a higher degree of flexibility in terms of space and time in implementation. The use of VR also allows the designers to incorporate a number of relevant real life scenarios that are useful for improving the social interaction competence of the ADHD children. There are some preliminary evidence of the usefulness of VR for training the ASD children, it's good to see the PI to propose the application of VR technology and training programs to enhance engagement and interaction in children with ADHD. This idea is original and novel and would certainly benefit the ADHD children, the families, and the society.

The study tries to examine and compare the effectiveness of VR and traditional training methods on changes of social interaction skills, inhibitions, emotional control, behavioural and emotional difficulties and conduct disorders together with a control condition. The PI has provided strong and sound arguments of making such a proposal to fill in the identified research gaps, e.g no previous study utilised social VR to assess social interaction competence and appropriateness in children with ADHD, and no randomised controlled trial (RCT) compared the effectiveness of social VR training and traditional social skills training. The proposed methodology of a three-arm RCT for this study is sound and will produce reliable and valid findings with appropriate statistical analyses.

Underneath are some queries, observations, and recommendations regarding to the methodology and analyses:

1. 105 participants will be recruited for the study. How's the planned gender mix of the participants?

2. It's stated in the proposal 'We will recruit 20 to 25 subjects per month so that the target sample size can be

recruited within a 6-month recruitment period.' Considering the trainings of 4 sessions per week for each participant, the PI may need to carefully evaluate there're sufficient time and space resources for execution of the research activities as planned. In particular contingencies need to be planned for data collection in this type of empirical studies during pandemic period.

3. It's stated in the proposal 'The sample size will be computed using G*Power, a statistical analysis tool, for the independent sample t-test [25]. A minimum of 34 subjects per group can provide 80% power to reject the null hypothesis with a medium effect size of 0.5 and a significance level of 0.05. 105 subjects (35 in each group) will be proposed for this study.' I'm not sure if it's good to have only independent sample t-test for analysis in this study. Many researchers and reviewers would have similar queries on the conducting too many individual t-tests. It's recommended to give strong justifications and references, if possible, for the adoption of 80% power and effect size of 0.5. Anyway, it's noted that ANCOVAs will be used as well in the later part of the proposal.

4. Incentives for a travel allowance for 105 participants ($10500), I think the proposed amount is too little considering the time and frequency of travel and participation. Also, there may be some attritions which add the need of increasing the budget for this item.

5. The PI has stated clearly all the ethical considerations as regards to the participants and parents.

6. The PI and the three Co-Is are very well experienced in the domain of proposed research areas and the application of the related technologies in similar experimental settings. They are fully capable of conducting the proposed research if granted.
